# Identification and Validation of a Cancer-Testis Antigen-Related Signature to Predict the Prognosis in Stomach Adenocarcinoma

**DOI:** 10.7150/jca.91842

**Published:** 2024-05-11

**Authors:** Geng Bian, Jie Cao, Weiyu Li, Dabing Huang, Xiping Ding, Xiaodong Zang, Yingquan Ye, Ping Li

**Affiliations:** 1 Department of Integrated Traditional Chinese and Western Medicine, Anhui Medical University, Hefei, Anhui 230022, China.; 2 Department of Chinese Integrative Medicine Oncology, The First Affiliated Hospital of Anhui Medical University, Hefei, Anhui 230022, China.; 3 Department of Respiratory, The First Affiliated Hospital of USTC, Division of Life Sciences and Medicine, University of Science and Technology of China, Hefei, Anhui 230001, China.; 4 National Clinical Research Center of Digestive Diseases, Beijing 100050, China.; 5 Liver Research Center, Beijing Key Laboratory of Translational Medicine in Liver Cirrhosis, Beijing Friendship Hospital, Capital Medical University, Beijing 100050, China.; 6 Department of Oncology, The First Affiliated Hospital of USTC, Division of Life Sciences and Medicine, University of Science and Technology of China, Hefei, Anhui 230001, China.; 7 Department of Geriatrics, The First Affiliated Hospital of USTC, Division of Life Sciences and Medicine, University of Science and Technology of China, Hefei, Anhui 230001, China.; 8 Gerontology Institute of Anhui Province, Hefei, Anhui, China; Anhui Provincial Key Laboratory of Tumor Immunotherapy and Nutrition Therapy, Hefei, Anhui 230001, China.; 9 Department of Pediatrics, The First Affiliated Hospital of USTC, Division of Life Sciences and Medicine, University of Science and Technology of China, Hefei, Anhui 230001, China.

**Keywords:** cancer-testis antigen, prognosis, immune microenvironment, biomarker, stomach adenocarcinoma

## Abstract

**Background:** Stomach adenocarcinoma (STAD) is the fifth most common cancer and the third leading cause of cancer-related deaths worldwide. Cancer-testis antigens (CTAs) participate in the pathogenesis and development of multiple cancers and are aberrantly overexpressed in various types of cancer. This study aimed to develop a CTA-related gene signature (CTARSig) to predict prognosis in STAD patients and explore its underlying mechanisms.

**Methods:** We performed differential and prognostic analyses of CTA-related genes and constructed a CTA-related signature (CTARSig) along with a novel nomogram to predict the prognosis of patients with STAD based on the Cox and The Least Absolute Shrinkage and Selection Operator. CTARSig was further validated in an external cohort (GSE84437). Additionally, univariate and multivariate Cox regression, as well as receiver operating characteristic (ROC) analyses, were performed to assess the CTARSig systematically. Single-sample gene set enrichment analysis and ESTIMATE were used to characterise the Tumor Immune Microenvironment (TIME) in patients with STAD. Furthermore, Gene Set Variation Analysis, Kyoto Encyclopedia of Genes and Genomes, and Gene Ontology analyses revealed the biological functions and signalling pathways associated with CTARSig. Finally, the human gastric cancer cell lines, HCG-27 and AGS, were used for *in vitro* and *in vivo* experiments, respectively, to further validate the role of ELOVL4.

**Results:** Eleven CTA-related genes were identified to construct the CTARSig. Kaplan-Meier curves, independent prognostic analysis, and ROC curves revealed that CTARSig could better predict survival in patients with STAD. Moreover, in our study, we demonstrated that ELOVL4 is upregulated in gastric cancer tissues and that its high expression is associated with poor survival. Additionally, *in vitro* and *in vivo* experiments demonstrated that ELOVL4 promotes the metastatic and invasive potential of STAD cells, suggesting it may be a potential therapeutic target for STAD.

**Conclusion:** In this study, a novel signature associated with CTAs was constructed for STAD, which may be a good predictor of patient prognosis. Thus, ELOVL4 may be a potential therapeutic target for gastric cancer. This study provides new insights into the potential roles of CTAs in gastric cancer.

## Introduction

Gastric cancer (GC), one of the most common malignant tumours of the digestive system, is associated with the highest mortality, poor prognosis, and multiple risk factors. It is the third most common cause of cancer-related deaths worldwide 1. Due to the poor outcomes associated with early diagnosis, the majority of patients with GC are diagnosed at advanced stages, missing the optimal surgical window. Currently, the primary treatment for advanced gastric cancer involves a combination of neoadjuvant chemoradiotherapy, molecular-targeted therapy, and immunotherapy. However, patients with GC still face a poor prognosis, especially in stomach adenocarcinoma (STAD) 2. Therefore, it is important to explore novel biomarkers for STAD to predict patient prognosis.

Previous studies have shown that numerous cancer-testis antigens (CTAs) are uniquely overexpressed in various types of cancer and are generally associated with poor prognosis. Several studies have demonstrated that CTA-related genes play a critical role in both cancer progression and effective immune responses in the tumour microenvironment 3. In recent years, numerous CTA-related genes have been shown to be highly expressed in multiple cancers, such as the melanoma-associated gene (MAGE-A) family, cancer/testis antigen-45 (CT45) family, and New York oesophageal squamous cell carcinoma 1 (NY-ESO-1), which is preferentially expressed in melanoma (PRAME). Additionally, various CTAs promote tumour initiation, metastasis, and drug resistance via different signalling pathways 4. A study in prostate cancer showed that SPAG9 promotes tumour growth and metastasis by regulating the JNK and mitogen-activated protein kinase (MAPKs) signalling pathways 5. In addition, KK-LC-1 promoted liver cancer progression by activating the Notch1 signalling pathway 6. However, the role of CTAs in STAD has not yet been fully explored.

This study aims to address this gap by constructing a CTA-related signature (CTARSig) based on the CTA-related genes in STAD. Through systematic validation, we investigate the potential of CTARSig as an independent prognostic factor for STAD. In addition, we validated the function of the CTARSig-related gene ELOVL4 in GC cells, further elucidating its potential role in STAD progression. Our findings are anticipated to pave the way for the development of targeted therapies in GC, offering potential improvements in treatment outcomes.

## Materials and Methods

### Data collection

Transcriptomic data and corresponding clinical parameters for patients with STAD were downloaded from The Cancer Genome Atlas (TCGA) repository. The TCGA-STAD cohort was designated as the training cohort for this study, with inclusion criteria requiring the availability of both survival time and status information, as well as complete transcriptome data. The transcriptomic data matrix and clinical parameters for the validation cohort (GSE84437) were downloaded from the Gene Expression Omnibus (GEO). A CTAs-related gene set was obtained from the Human Genome Database.

### Ethical statement

All the study designs and experiments were approved by the Ethics Committee of the First Affiliated Hospital of the University of Science and Technology of China (USTC) (Agreement Number: 2019-X(H)-001). This study was conducted in accordance with the principles of the Declaration of Helsinki.

### Identification of CTAs-related genes in STAD

The R package “limma” was used to obtain expression data for CTAs-related genes in the TCGA-STAD cohort and to further determine differentially expressed genes (DEGs) between tumour and normal tissue (fold change > 2, false discovery rate (FDR) < 0.05). The “pheatmap” package was used to visualise DEGs volcanoes and expression heat maps. Additionally, the R packages “limma” and “sva” were used to extract the expression matrices of DEGs from both the TCGA cohort and GEO cohort, identify the intersecting genes between the two cohorts, and perform batch correction on the expression matrices of intersecting genes from both cohorts to generate DEGs expression matrices. Furthermore, mRNA expression and survival data were integrated for subsequent analyses. Finally, univariate Cox regression analysis was performed using the 'survival' and 'survminer' packages to identify survival-related CTAs-related genes in the TCGA cohort (*p* < 0.05).

### Establishment of a CTARSig in STAD

Firstly, univariate Cox regression analysis was performed using the 'survival' and 'survminer' packages to identify survival-related CTAs-related genes in the TCGA cohort (*p* < 0.05). To prevent overfitting, the least absolute shrinkage and selection operator (LASSO) algorithm was applied to survival-associated genes to screen for the optimal genes involved in the construction of CTA-related signatures 7,8. This process was realised with the R packages 'glmnet' and 'survival'. The risk score for each patient was derived from the expression values of the genes in CTARSig and their corresponding regression coefficients. The risk equation is:



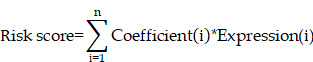



The patients were divided into high- and low-risk groups based on their median risk scores.

### Validation of the CTARSig in STAD

Firstly, we performed a survival validation across different cohorts. The R package 'pheatmap' was used to create the expression heat map of signature genes in TCGA, GEO, and merged cohorts. Subsequently, the R packages' survivor' and 'survminer' were used to plot the risk score rankings, survival status maps, and Kaplan-Meier (K-M) curves for the three cohorts. Univariate and multivariate Cox regression analyses were then performed to assess whether CTARSig was an independent prognostic factor of STAD. Finally, time-dependent receiver operating characteristic (ROC) curves were generated using 'survminer', 'survival' and 'timeROC' to assess the prognostic efficacy of CTARSig.

### Nomogram construction

To better predict clinical outcomes in patients with STAD, we combined patient risk status based on CTARSig with selected clinicopathological parameters to construct a nomogram. Based on the results of multivariate Cox regression analysis, patient age, tumour grade, TNM stage, and risk status were used to construct a nomogram. In addition, calibration curves for the Hosmer-Lemeshow test were drawn to evaluate the predicted and actual outcomes (method = 'boot', B = 1000). The 'survival' ', regplot', and' rms' packages were applied in this process.

### Correlation of the CTARSig and the tumour immune microenvironment

The CIBERSORT algorithm is used to characterise and quantify different types of immune cells with high throughput 9. The fraction of 22 tumour-infiltrating immune cells (TIICs) was determined using “CIBERSORT”, “parallel”, “limma”, “e1071”, and “preprocessCore.” Subsequently, differences in TIICs between the two subgroups were determined.

Gene set enrichment analysis (GSEA) allows the analysis of gene sets with physiological and biological functions 10. Single sample GSEA (ssGSEA) is an evolution of GSEA for analysing the absolute enrichment of a gene set 11. The “GSEABase” and “GSVA” packages were applied to the ssGSEA analysis to obtain immune cell scores and immune-related function scores for each sample. Furthermore, the “limma” package was used to analyse differences in scores across risk groups.

ESTIMATE is an algorithm used for determining tumour purity based on expression information 12. The ESTIMATE score encompasses both the total immune and stromal fractions, which are inversely related to tumour purity. In this study, the “ESTIMATE” package was used to calculate the stromal cell score and immune cell score for each STAD case's tumour tissue. Finally, the “ggpubr” package was used to plot box plots illustrating the variability of the different scores within the high- and low-risk groups.

### Gene ontology (GO) and gene set variation analysis (GSVA)

The DEGs (fold change > 2, FDR < 0.05) between the two risk groups were determined with “limma”. Subsequently, the R packages “clusterProfiler”, “enrich”, “GOplot”, “ggplot2”, “enrich”, “GOplot”, and “org. Hs. e.g. db” were used to perform GO analysis of DEGs and explore their enrichment in molecular function, biological processes, and cellular components.

GSVA was used to detect differences in pathway activity among the sample populations 13. Specifically, GSVA was used to assess the enrichment of the Kyoto Encyclopedia of Genes and Genomes (KEGG) pathways in the two risk groups. Furthermore, the correlation between the signature-related gene expression and pathways was analysed. This analysis was performed using the R packages “reshape2”, “limma”, “GSVA”, “ggplot2”, and “GSEABase”.

### Analysis of Clinical Therapeutic Drug Sensitivity

To explore the potential significance of CTARSig in guiding individualised clinical treatment, we used the R package “pRRophic” to obtain half-maximal inhibitory concentrations (IC50) for different drugs across the risk groups. Subsequently, “ggpubr” was used to create box plots illustrating variability across drugs (*p* < 0.001).

### Clinical tissue collection

GC tissue samples were collected from patients diagnosed at the First Affiliated Hospital of the USTC between 2019 and 2021. Patients who had received any preoperative therapy, as well as those with multiple primary GC or hereditary GC, were excluded. All patients provided written informed consent for the clinical use of their tumour tissues.

### Cell culture and treatments

Five human gastric cancer cell lines (HCG-27, MKN-74, AGS, N87, and MKN-45) and the normal human gastric cell line GES-1 were purchased from the American Type Culture Collection. All human gastric cell lines were stored at the Anhui Provincial Key Laboratory of Tumor Immunotherapy and Nutrition Therapy. AGS cells were grown in F12K medium (Ham's F-12 Medium, Gibco, USA) containing 10% FBS, while the other cell line was cultured in RPMI 1640 medium (RPMI 1640, Gibco, USA) supplemented with 10% foetal bovine serum (FBS, Gibco, USA) and 2% penicillin/streptomycin (Trans Gen Biotech, Beijing, China).

### RNA interference

For transfection treatment, HCG-27 and AGS cells were seeded in a 6 cm dish 24 h before transfection with the siRNA-negative control (NC) and ELOVL4-siRNA groups. Cells were treated with 5 µl of siRNA, and GenMute™ Reagent provided by SignaGen Laboratories (Baltimore, USA) was diluted in dilute GenMute™ Transfection Buffer working solution (1x) for 5 min. The samples were then mixed and incubated at room temperature for 15 min before being added to a cell culture plate. After transfection for 48-72 h, the cells were collected for subsequent experiments. Two siRNA specific for ELOVL4 were used in this study, with the following sequences: siNC: UUCUCCGAACGUGUCACGUTT, ACGUGACACGUUCGGAGAATT; siELOVL4-1: GGACAUACAAAGAGCCUAATT, UUAGGCUCUUUGUAUGUCCTT; siELOVL4-2: GUCUAGUGCUCAUUAUCUATT, UAGAUAAUGAGCACUAGACTT.

### Lentivirus packaging and transfection

This method was conducted as previously described 14. Human ELOVL4 short hairpin RNA (shRNA) was obtained from Genomeditech Co., Ltd. (Shanghai, China). Briefly, HEK293T cells were co-transfected with the viral packaging plasmids pSPAX2 and pMD2. G, along with control shRNA or shRNA against ELOVL4. After 48 h of transfection, the lentiviral supernatants were harvested and filtered. HCG-27 cells were infected with virus particles in addition to 10μg/mL Polybrene. Stable cells were screened using puromycin (2μg/mL) and confirmed by the western bolt with anti-ELOVL4 antibody. The following target sequences were used: sh-ELOVL4#1:5-GCTCTAGTAACCTGAATAA-3, sh-ELOVL4#2:5-GGGCAGTTGATGGCAAGTA-3, sh-ELOVL4#3:5-GCCTCCCACTAAACATCAT-3.

### Western blotting (WB)

Total protein was extracted from cells and tissues using a radioimmunoprecipitation assay buffer. Nuclear and cytoplasmic protein extracts from the cells and controls were prepared using nuclear and cytoplasmic protein extraction kits. The protein concentrations were determined using BCA kits and heated at 97°C for 10 min. The heated proteins were separated using 10% sodium dodecyl sulfate-polyacrylamide gel electrophoresis and transferred to polyvinylidene difluoride (PVDF) membranes. The membranes were then incubated for 15 min at room temperature with a protein-free rapid-blocking buffer. Subsequently, the blots were incubated with ELOVL4 and GAPDH primary antibodies at 4°C overnight. After washing with TBST, the membranes were hybridised with an appropriate secondary antibody at room temperature for 1 h. Finally, images of the WB bands were obtained using ChemiCapture, and the intensity in each group was measured using ImageJ software. GAPDH was used as an internal control.

### Quantitative real-time PCR

We used the InnuPREP RNA Mini Kit (Berlin, Germany) to extract total RNA from cells, which was then reverse transcribed to synthesise cDNA using the Trans-Script All-in-One First-Strand cDNA Synthesis Super Mix kit (Trans Gen Biotech, Beijing, China) according to the manufacturer's instructions. All target gene mRNA levels were quantified using Trans Start Top Green qPCR Super Mix (Trans Gen Biotech) on a Light Cycler 96 PCR instrument system (Roche). Using the 2^-ΔΔCt^ method, we normalised target gene expression levels to GAPDH expression level before comparing the relative levels of the target genes. The primer sequences of ELOVL4 are as follows: Forward (5′-3′): ACTAGGGCTGACTGCGTTC, Reverse (5′-3′): GGGCAGTCGGTGTAGAGAGA.

### Proliferation assay and colony formation

For the CCK8 assay, cells were seeded and cultured in 96-well plates at a density of 2,000 cells/well. After incubation for 0, 24, 48, 72, or 96 h, CCK-8 reagent was added to each well, and the absorbance was determined at 450 nm. Additionally, 1000 cells/well were plated in 6-well plates and cultured in a medium containing 10% FBS and 2% penicillin/streptomycin. The medium was changed every 2-3 days. After 10 d, if colonies were observed, the plates were washed with PBS, and the cells were fixed and stained with crystal violet for 20 min.

### Invasion and migration assay

The invasive potential of the GC cells was measured using Matrigel (BD, Franklin Lakes, NJ, USA) and trans well inserts (8.0 μm, Costar, Manassas, VA, USA) containing polycarbonate filters with 8-μm pores. The inserts were coated with 50 μl of 1 mg/ml Matrigel matrix according to the manufacturer's instructions. Images were acquired using a fluorescence microscope and quantified using the ImageJ software.

### Immunohistochemical (IHC) staining

IHC staining of patient and nude mouse tissues was performed following standard protocols 14. As previously described, IHC staining was performed on patient and mouse tumour tissues using anti-Ki67 and anti-ELOVL4 antibodies. Following immunohistochemical scoring, the intensity of staining (0 = negative, 1 = weak, 2 = moderate, 3 = strong) and the percentage of positively stained tumour cells (1 = 0-25%, 2 = 26-50%, 3 = 51-75%, 4 = 75-100%) were used for quantification. Two pathologists independently assessed the data.

### Mouse xenograft models

In brief, BALB/c nude mice (5-6 weeks old male) were randomly grouped and injected subcutaneously with 2×10^7^sh-Control, sh-ELOVL4 HCG-27 cells. The tumour volume was measured every 5 d for 30 d and calculated using the following formula: volume = (Length × Width2)/2. After 30 d, the tumour tissues were weighed, and haematoxylin and eosin HE and IHC staining were performed to identify tissue sections expressing Ki-67 and ELOVL4. All the experiments were approved by the Animal Ethics Committee of the USTC (No.2024-N(A)-81).

### Statistical analysis

All experiments were repeated thrice. Statistical analyses were conducted using R software (v4.1.0) and SPSS 23.0, and the results were visualised using GraphPad Prism 8.0 (V8, USA). Student's t-test (two-tailed) or one-way ANOVA was used to compare the means of two or three groups. Statistical significance was set at *p* < 0.05.

## Results

### Identification of CTAs-Related genes in STAD

**Figure 1:**
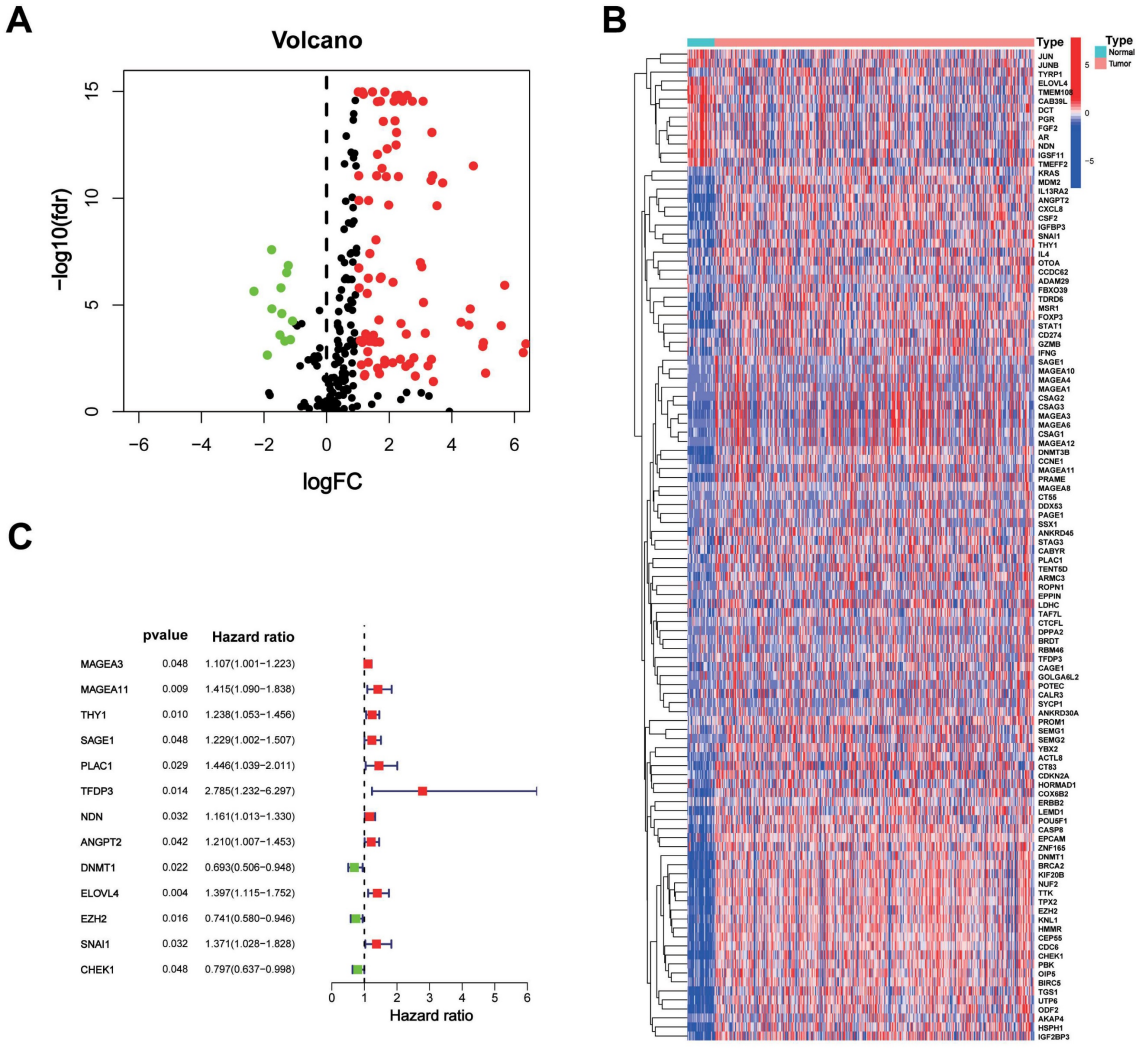
**CTAs-related genes in STAD. (A)** The volcano plot of 110 DEGs. **(B)** Heat map of expression of the DEGs in normal and tumour samples.** (C)** The risk forest plot showed that 13 CTAs-related genes were associated with the prognostic risk of STAD.

The study included data from the TCGA database comprising 375 STAD tumours and 32 normal samples. Additionally, data from the GeneCards database, consisting of 398 CTA-related genes with correlation scores greater than 3, were included. Among these genes, 110 were identified as DEGs between tumour and normal tissues, with 12 being downregulated genes (Figure 1A). The heat map displays the expression of these DEGs between the tumour and normal tissues (Figure 1B).

### CTARSig in STAD

**Figure 2:**
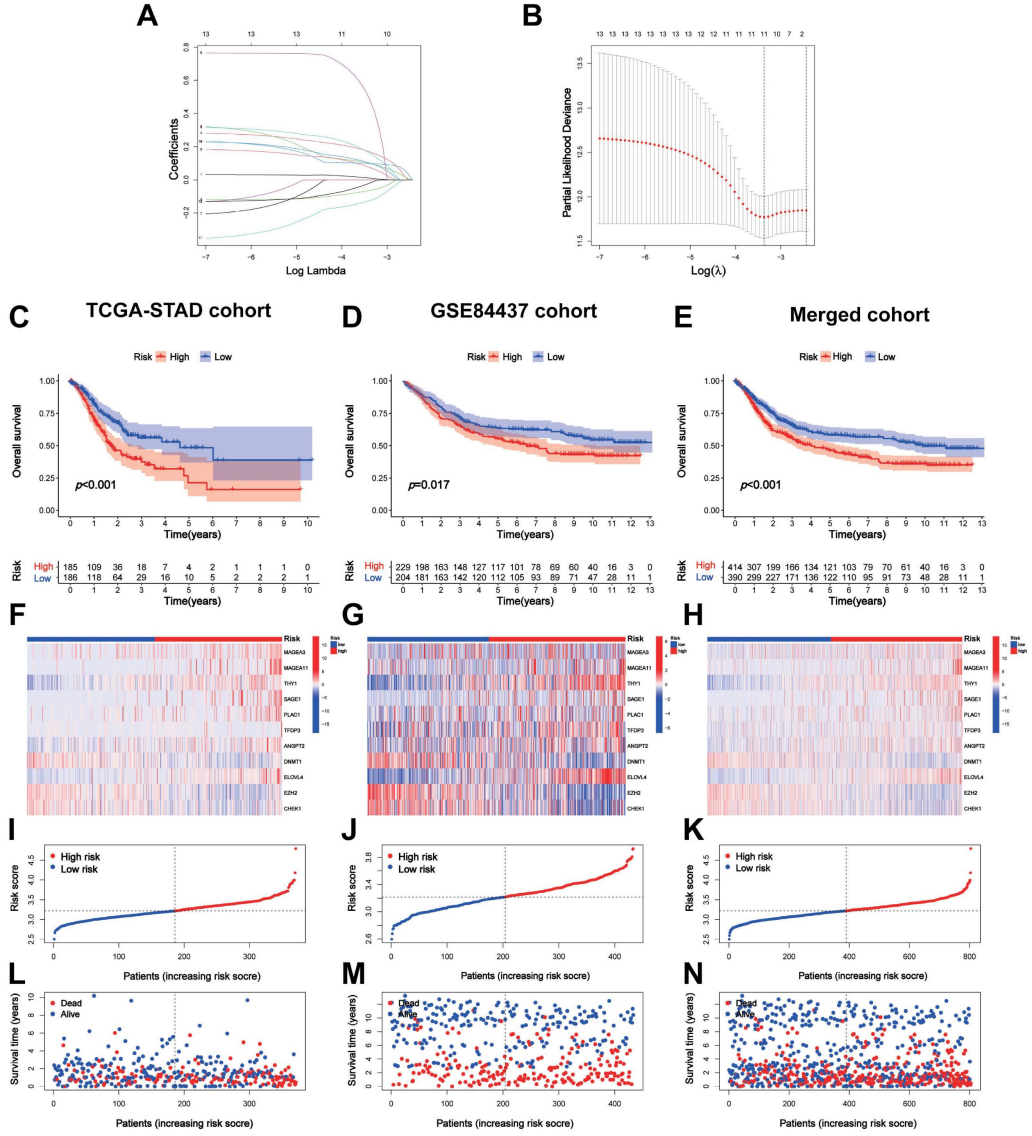
**Construction and validation of** CTARSig**. (A-B)** The coefficient and partial likelihood deviance of the CTARSig.** (C-E)** K-M curves for OS in the TCGA, GEO, and merged cohorts. **(F-H)** Heat map of expression of the 11 CTAs-related genes in the three cohorts. **(I-K)** Risk score distribution in the three cohorts. **(L-N)** Survival time and status in the three cohorts.

A total of 371 patients in the TCGA-STAD cohort contained clinicopathological parameters, survival information, and complete transcriptomic data (Table 1). Univariate Cox regression analysis revealed that the 13 CTA-related genes were associated with the prognostic risk of STAD (Figure 1C). To avoid overfitting, we performed LASSO regression analysis (Figure 2A, B) and identified 11 optimal genes to construct CTARSig (Table 2). The risk score for each patient was calculated using the risk formula from the signature. Risk score = MAGEA3 × (0.010700239) + MAGEA11 × (0.167350315) + THY1 × (0.097880794) + SAGE1 × (0.100101094) + PLAC1 × (0.158666022) + TFDP3 × (0.475956093) + ANGPT2 × (0.073519232) - DNMT1 × (0.063247504) + ELOVL4 × (0.098339527) - EZH2 × (0.12369429) - CHEK1 × (0.018454819). Based on the median risk score of the TCGA cohort, we divided all patients into high- and low-risk groups.

### Validation of the CTARSig in STAD

We validated CTARSig in TCGA, GEO, and merged cohorts. The K-M curves indicated that patients in the high-risk group in all three cohorts had a significantly worse prognosis than those in the low-risk group (Figure 2C-E). The heat map displays the expression status of 11 CTA-related genes in CTARSig in the three cohorts (Figure 2F-H). The risk score distribution and survival status plots showed an increase in the proportion of patients with mortality as their risk score increased (Figure 2I-N).

### Evaluation of the CTARSig in STAD

**Figure 3:**
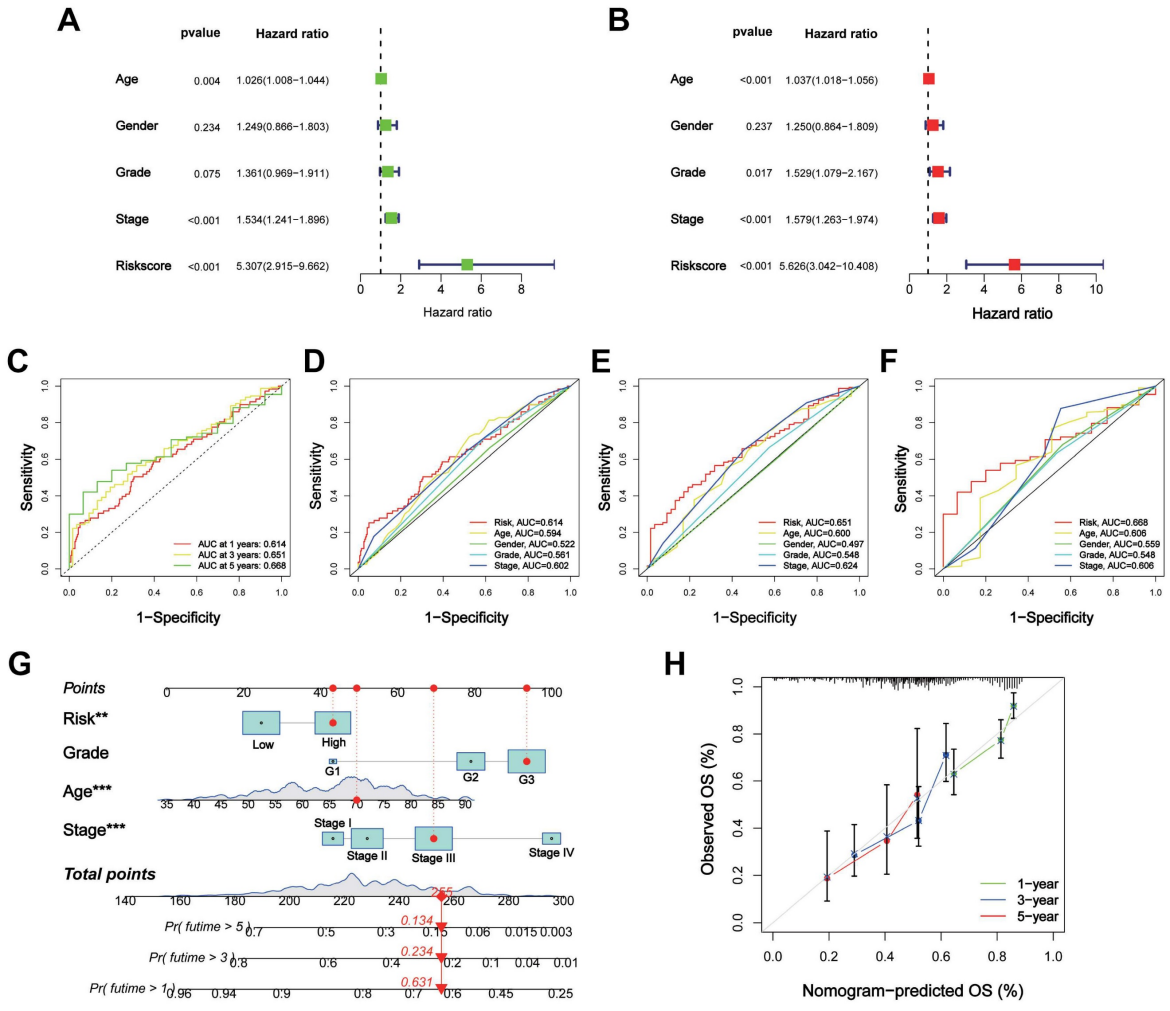
** Assessment of the CTARSig in STAD**. **(A-B)** Forest plot for uni- and multi-Cox regression. **(C)** ROC curves of 1-, 3-, and 5-year survival for the CTARSig. **(D-F)** Comparison of the prediction accuracy of the CTAs-related signature with age, gender, stage and grade in 1-, 3-, and 5-year. **(G)** Nomogram for predicting survival.** (H)** The calibration curves of the nomogram. *p < 0.05, **p < 0.01, and ***p < 0.001.

Univariate and multivariate Cox regression analyses indicated that CTARSig was an independent prognostic risk factor for STAD (all *p* < 0.001) (Figures 3A, B). In addition, patient age and tumour stage were independent risk factors.

ROC curves were generated to assess the prognostic predictive efficacy of CTARSig. The area under the curve (AUC) values for CTARSig predicting overall survival (OS) at 1-, 3- and 5-year were 0.614, 0.651, and 0.668, respectively (Figure 3C). In addition, the AUC values for CTARSig at 1-, 3- and 5-year were higher than those for age and stage (Figure 3D-F).

### Nomogram construction and validation

According to the Cox regression analysis, age, grade, stage, and risk score demonstrated the impact of CTARSig on the prognosis of STAD. Therefore, we constructed a nomogram based on these variables to predict the prognosis of patients with STAD (Figure 3G). The corresponding scores for each factor were calculated using a nomogram, and the total score served as a predictive tool for prognosis. The calibration curve demonstrated good consistency between the nomogram-predicted survival and the actual situation (Figure 3H). Furthermore, the ROC curves indicated that the nomogram had a better prognostic predictive efficacy than the other clinicopathological parameters (Figure S1A). Cox regression analysis suggested that the nomogram was an independent prognostic factor of STAD (Figure S1B).

### Correlation of the CTARSig with TIME in STAD

**Figure 4:**
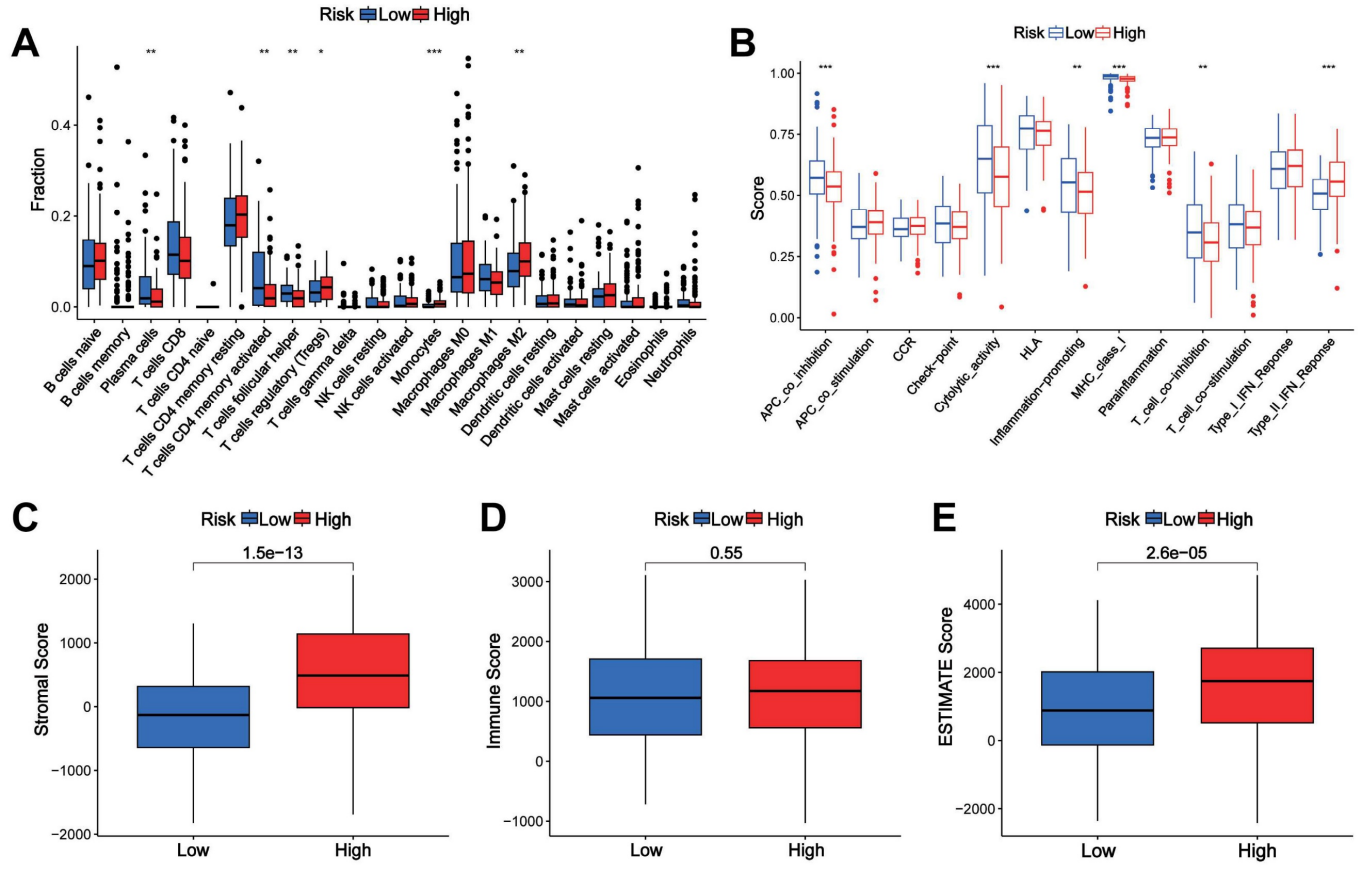
**Association of the CTARSig with TIME in STAD. (A)** Differences in immune cells between the high- and low-risk groups based on CIBERSORT. **(B)** Differences in immune-related functions between the high- and low-risk groups based on ssGSEA. **(C-E)** Stromal score, immune score, and ESTIMATE score in the high- and low-risk groups. *p < 0.05, **p < 0.01, and ***p < 0.001.

The CIBERSORT algorithm showed differences in plasma cells, activated memory CD4+ T cells, follicular helper T cells, regulatory T-cells, monocytes, and M2 macrophages between the high- and low-risk groups (Figure 4A). The ssGSEA results revealed significant differences in immune-related functions such as APC co-inhibition, cytolytic activity, inflammation promotion, MHC class I and T cell co-inhibition, and type II IFN response between the high-risk and low-risk groups (Figure 4B). The stromal and ESTIMATE scores were significantly higher in the high-risk group, while the immune scores did not differ between the two groups (Figures 4C-E).

### GO and GSVA analysis of the CTAs-related signature CTARSig in STAD

**Figure 5:**
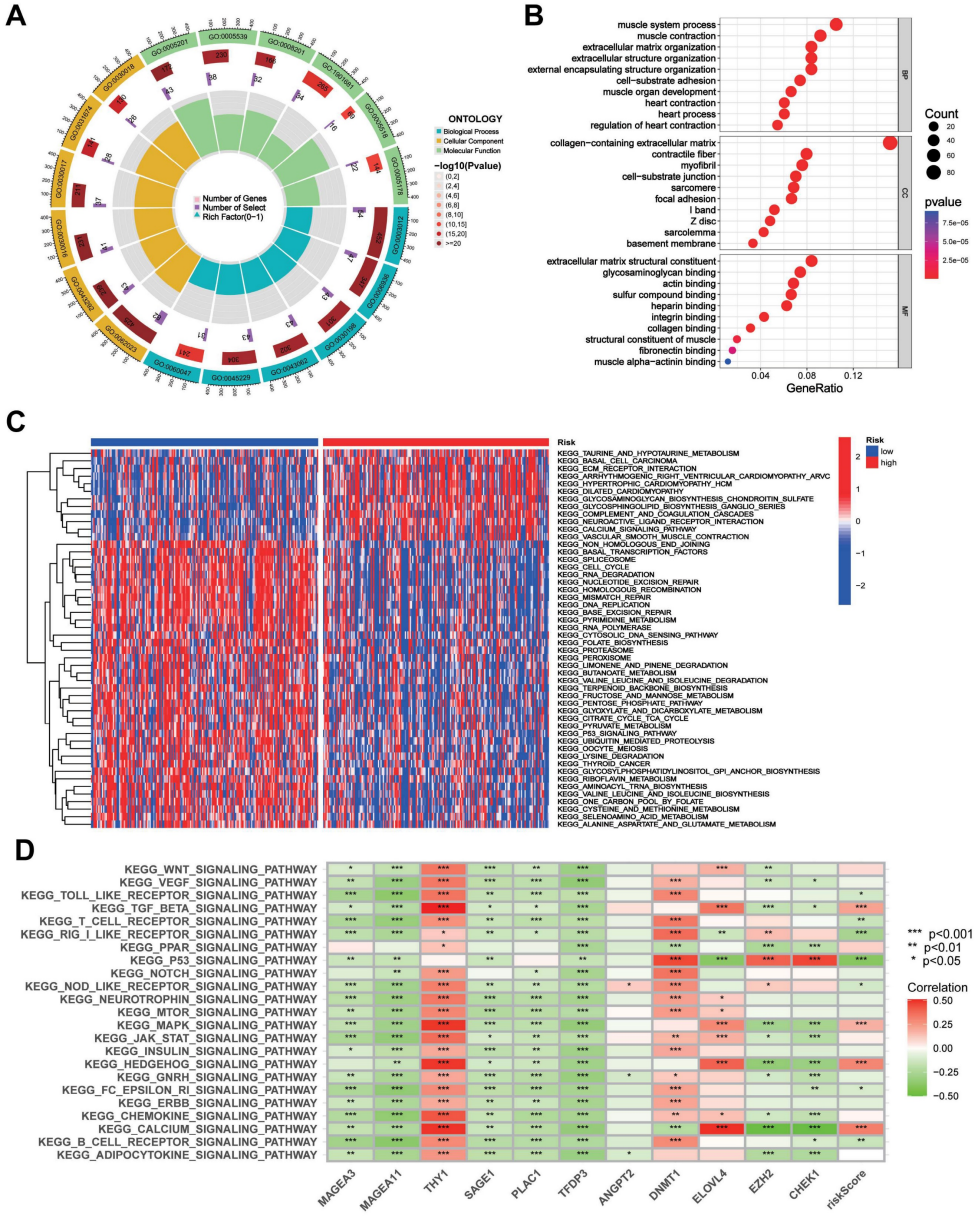
**GO and GSVA**. **(A, B)** GO analysis indicated the enrichment of DEGs between the high- and low-risk groups. **(D)** Heat map of functional pathway enrichment differences between the two risk groups based on GSVA. **(E)** Heat map of the correlation between the expression of signature genes and signalling pathways.

GO analysis showed that DEGs were enriched in biological processes such as muscle system processes, muscle contraction, extracellular matrix organisation, and extracellular structure organisation. In terms of cell composition, DEGs were enriched in the collagen-containing extracellular matrix, contracting fibres, myofibrils, and cell-substrate junctions. In terms of molecular function, DEGs were enriched in extracellular matrix structural constraints, glycosaminoglycan binding, actin binding, and sulfur compound binding (Figures 5A, B).

GSVA indicated that taurine and hypotaurine metabolism, basal cell carcinoma, ECM receptor interactions, and calcium signalling pathways were enriched in the high-risk group. Meanwhile, the cell cycle, RNA degradation, mismatch repair, DNA replication, P53 signalling pathway, and pyrimidine metabolism were enriched in the low-risk group (Figure 5C). The heatmap demonstrated a broad correlation between the expression of 11 genes in CTARSig and the signalling pathways associated with tumour evolution (Figure 5D).

### Prediction of drug sensitivity by the CTARSig in STAD

**Figure 6:**
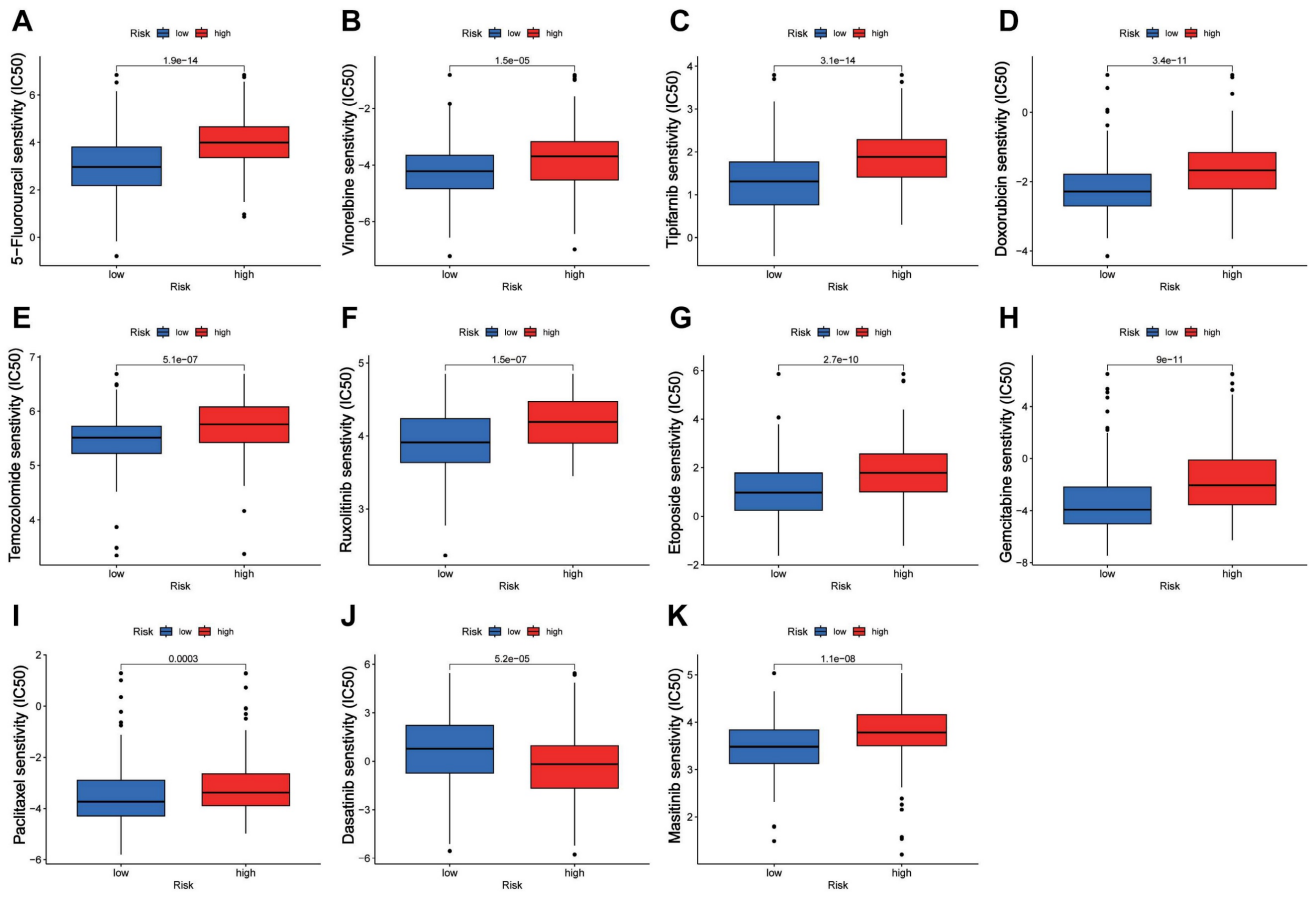
** Drug sensitivity analysis. (A-K)** Boxplots of IC50 values for different agents in low-risk and high-risk groups.

Drug sensitivity analysis showed that the IC50 values of 5-fluorouracil, vinorelbine, tipifarnib, doxorubicin, temozolomide, ruxolitinib, etoposide, gemcitabine, paclitaxel, and masitinib were significantly lower in the low-risk group than in the high-risk group, whereas the opposite result was observed for dasatinib (Figure 6A-K).

### Knockdown ELOVL4 Inhibits Malignant Phenotype of GC cell

**Figure 7:**
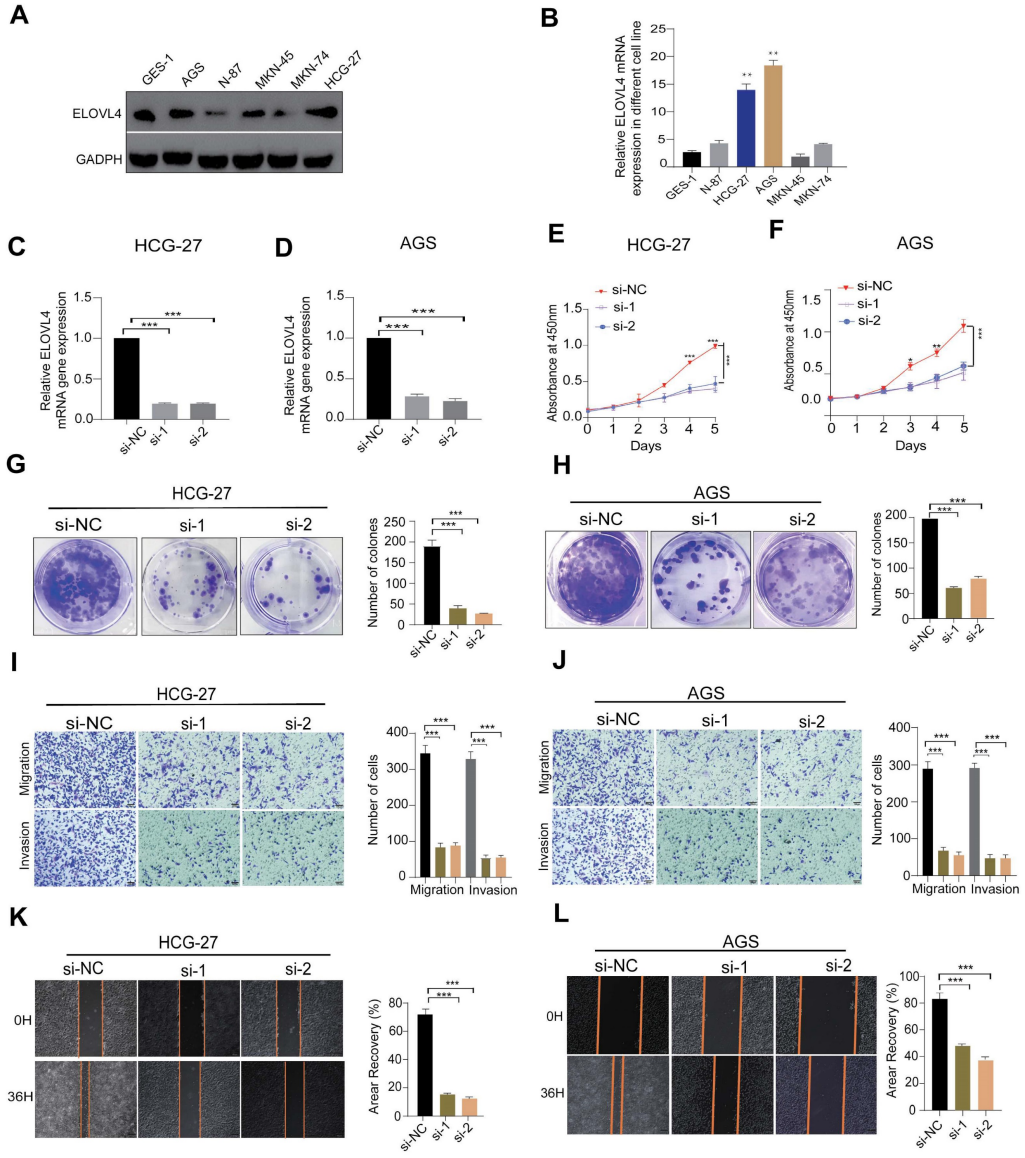
** ELOVL4 Promote the metastatic and invasion potential of GC cells (A, B)** Relative protein and mRNA expression of ELOVL4 in five GC cell lines Compared with normal GC cell GES-1as detected by western blot and RT-qPCR analysis. **(C, D)** RT-qPCR analysis of ELOVL4 expression efficacy by ELOVL4 Knockdown in the GC cells transfected with siRNA-NC and ELOVL4-siRNA groups. **(E, F)** Cell viability assesses the silencing of ELOVL4 by CCK-8 at different time points: 24, 48, 72, and 96h. **(G, H)** Representative images of colony formation assay in transfected cells (right panel) and quantitative results (left panel). **(I,J)** Representative data from Transwell migration and Matrigel invasion assays were obtained with the ELOVL4 knockdown cells.** (K, L)** Representative data from wound healing migration assays were obtained with the ELOVL4 knockdown cells. Data are expressed as mean±SD (*p < 0.05, **p < 0.01, and ***p < 0.001.) in normal gastric cell GES-1.

Among the 11 CTA-related genes involved in the signature construction, the role of ELOVEL4 in STAD remains unclear. To verify the effects of ELOVEL4 on cell proliferation, migration, and invasion in gastric cancer, we analysed the relative expression levels of ELOVL4 in five GC cell lines (MKN-45, HCG-27, MKN-74, N87, and AGS) and GSE-1 cells. The expression level of ELOVL4 in the HCG-27 and AGS cell lines was higher than that in the other cell lines (Figure 7A-B). Consequently, we selected the HCG-27 and AGS cell lines for further experiments. We then used siRNA to successfully interfere with the mRNA expression of ELOVL4, and the efficiency of ELOVL4 knockdown was detected by RT-qPCR in both cell lines (Figure 7C-D). After silencing ELOVL4 expression, the colony-forming assay results showed significant inhibition of cell viability (Figure 7G-H). Subsequently, a CCK8 assay was performed to assess the effect of si-ELOVL4 on the proliferation of AGS and HCG-27 cells (Figure 7E-F). Moreover, to further detect whether ELOVL4 affects the invasion and migration of GC cells, the transwell assay revealed that HCG-27 and AGS cells exhibited markedly decreased invasion upon ELOVL4 knockdown (Figure 7I-J). The results of the wound healing assay demonstrated that knockdown of ELOVL4 significantly decreased the migration of HCG-27 and AGS cells (Figures 7K-L).

### Higher ELOVL4 expression predicts poor prognosis and inhibits ELOVL4 suppresses GC tumorigenesis *in vivo*

**Figure 8:**
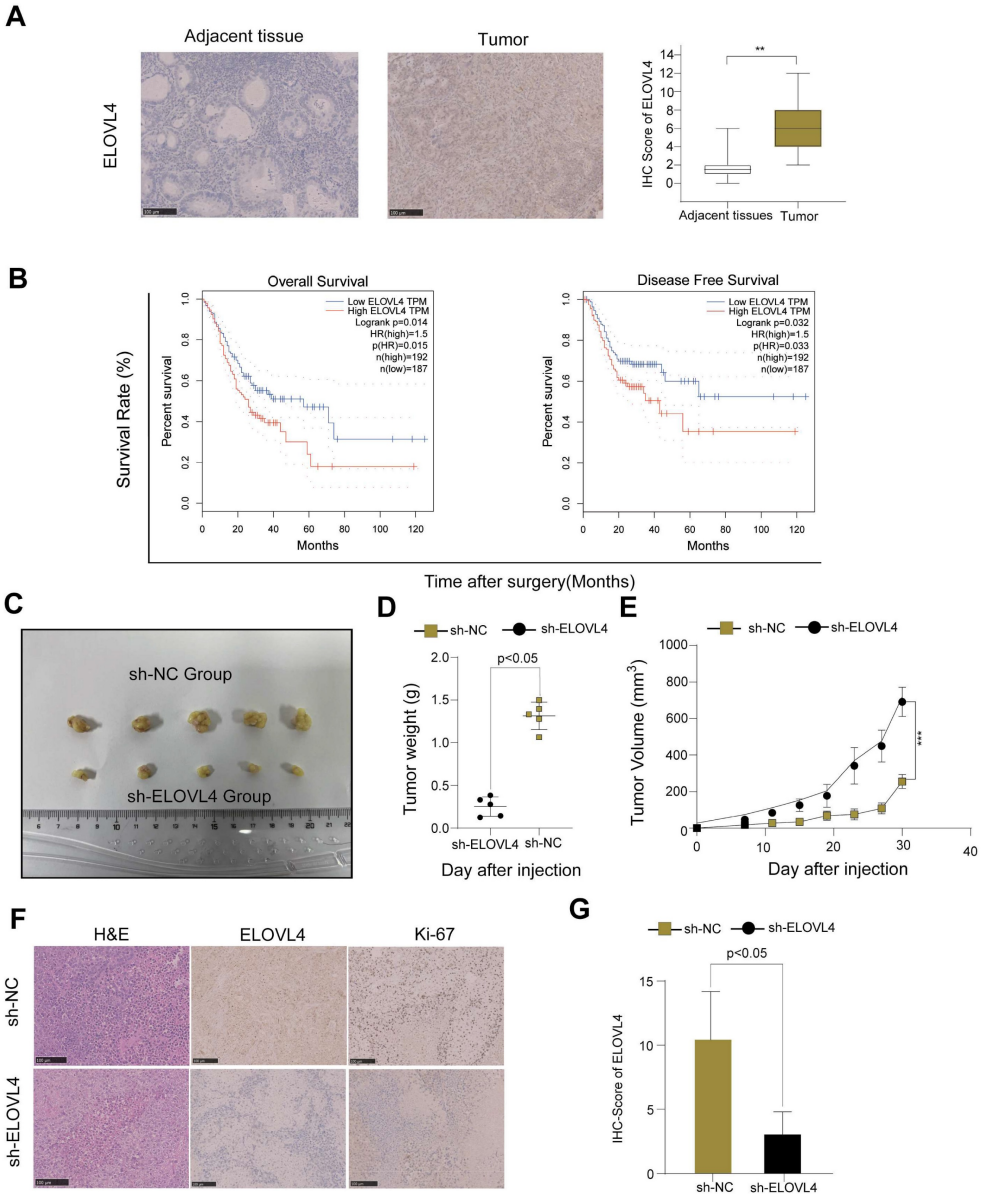
** ELOVL4 had high expression in GC tissues and promoted GC cell growth* in vivo*. (A)** Representative images of immunohistochemistry staining of ELOVL4 protein expression with GC tissues and normal tissues. **(B)** The relationship between ELOVL4 expression and overall survival or disease-free survival by using the website GEPIA 2.** (C)** Subcutaneous xenograft tumour images and tumour growth of HCG-27 cells with ELOVL4-NC and ELOVL4-KD in nude mice.** (D-E)** The gross images and the tumour weight of nude mice with ELOVL4-NC and ELOVL4-KD Group. **(F)** Representative photomicrographs of HE staining and IHC staining of ELOVL4, Ki-67 in GC nude mice tissues with ELOVL4-NC and ELOVL4-KD Group. (Magnification: ×200). **(G)** IHC score analysis of ELOVL4 expression in nude mice tissues with ELOVL4-NC group revealed a significant difference compared to the ELOVL4-KD Group (*p* < 0.05).

To confirm whether ELOVL4 was indeed upregulated in GC, we examined a small cohort of 46 paired GC samples using IHC analysis. Therefore, an IHC assay using samples from 46 patients with GC showed higher ELOVL4 expression than that in paired adjacent tissues (Figure 8A). As shown in Figure 8B, high ELOVL4 expression was associated with shorter overall survival (OS) and Disease-Free Survival (DFS) in gastric cancer patients (*p* < 0.05). Furthermore, to explore whether ELOVL4 facilitates tumorigenesis in vivo, we generated HCG-27 cells stably transfected with sh-Control or Sh-ELOVL4 and performed a subcutaneous xenograft experiment in nude mice (Figure 8C). Mice inoculated with sh-ELOVL4 cells exhibited slower tumour growth and lower weight than those inoculated with sh-control cells (Figure 8D-E). In addition, IHC results showed that tumours dissected from the sh-NC group exhibited higher ELOVL4 staining than those dissected from the sh-ELOVL4 group. IHC analysis confirmed that sh-ELOVL4 inhibited the expression of the proliferation marker Ki-67 (Figure 8F-G). These results, taken together, strongly support the idea that ELOVL4 overexpression leads to gastric cancer progression* in vitro* and *in vivo*.

## Discussion

GC is the most common malignant human cancer and ranks among the top five morbidities and mortalities worldwide 15. Studies have reported a 5-year survival rate of > 90% for early-stage GC; however, early diagnosis remains limited, with most patients diagnosed at an advanced stage 16. Recently, the combination of chemotherapeutic and targeted therapies as first-line therapy for advanced GC has improved clinical efficacy, but the prognosis has not substantially improved 17. Hence, there is an urgent need to identify specific biomarkers to determine the prognosis of patients with GC.

Previous studies have indicated that CTA-related genes are overexpressed in many types of cancers. Several studies have revealed that a number of CTAs regulate the epithelial-mesenchymal transition (EMT) and enhance cancer stem-like cells, promoting metastasis, invasion, and tumourigenesis, which are associated with poor prognosis in patients with cancer 18-20. In this study, we constructed a novel CTARSig gene based on the CTA-related genes. Validation using different methodologies demonstrated that the risk scores based on CTARSig were STAD-independent prognostic factors with good predictive finiteness and stability.

Among the 11 CTA-related genes involved in the construction of CTARSig, MAGEA3 promotes the proliferation of GC cells and chemotherapy drug resistance 21 and is associated with lymph node metastasis and immune infiltration in GC 22. In contrast, the upregulation of MAGEA11, a member of the MAGE family, in GC has been shown to be significantly associated with lower survival rates and immune infiltration, suggesting that MAGEA11 may be a potential biomarker and therapeutic target in GC 23. THY1 is a risk factor for CTARSig, and previous studies have shown that patients with GC and high THY1 expression exhibit lower overall survival (OS) 24. *In vitro* experiments have shown that THY1 knockdown inhibits the proliferation and migration of GC cells while increasing autophagy 24. Another signature risk factor, placenta-specific 1 (PLAC1), has been confirmed to be associated with AKT/GSK-3 β/ Cyclin D1 signalling pathway, promoting cell proliferation in GC 25. TFDP3 has been confirmed to be associated with epistemic mesenchymal transition in GC and is significantly correlated with the prognosis of patients 26.

Additionally, ANGPT2 overexpression in GC is associated with a poor prognosis, and ANGPT2 can predict immune therapy and chemotherapy responses in GC 27. DNMT1 expression is associated with gastric carcinogenesis and partially dictates 5-Azacytidine sensitivity and RAS/MEK/ERK activity in GC cells 28,29. Previous research has shown that EZH2 upregulation may be associated with advanced TNM stage and poor prognosis in GC, indicating that combining EZH2 inhibitors and STAT3 knockdown may be a potential therapy for GC 30. Furthermore, the targeted inhibition of EZH2 expression can effectively improve the tolerance of GC cells to chemotherapeutic agents 31. Moreover, it has been demonstrated that reduced CHEK1 mRNA expression is an unfavourable prognostic variable in patients with GC 32. However, the role of ELOVL4 in CTARSig as a risk factor for GC remains unclear.

ELOVL4, a newly identified CTAs-related gene, has been explored in several tumours, including neuroblastoma 33, squamous cell carcinoma 34, bladder cancer 35, glioblastoma multiforme (GBM) 36. Based on our in vitro experiments, we found that siRNA-mediated inhibition of ELOVL4 suppressed the proliferation of HCG-27 and AGS cells, as confirmed by CCK-8, colony formation, and EdU assays. A wound-healing assay shows that knockdown ELOVL4 reduce GC migration ability. Transwell experiments revealed that silencing of ELOVEL4 inhibited GC cell invasion, indicating that downregulation of ELOVL4 significantly inhibited GC cell invasion and progression. In addition, ELOVL4 is upregulated in GC, and higher ELOVL4 expression predicts a poorer prognosis; inhibition of ELOVL4 suppresses GC tumorigenesis in vivo. These results, taken together, demonstrate that ELOVL4 is a potential therapeutic target for gastric cancer.

The tumour immune microenvironment (TIME) plays a key role in identifying immune modifiers of cancer progression and developing cancer immunotherapies 37. In addition, previous studies have indicated that upregulated or downregulated genes in different types of cancers can induce immune cells 38-41. Several studies have indicated that the microenvironment plays important roles in immune escape and resistance to cancer treatments, leading to the progression of malignant tumours. In our study, we investigated immune cell infiltration in GC tissues under various risk variables. Based on this signature, we found higher levels of monocyte and M2 macrophage infiltration in STAD tissues in a high-risk population, which provides a new idea to further investigate immune cell infiltration in STAD tissues. In addition, we analysed relevant immune function assessments for signature risk stratification and found that type II IFN responses were significantly higher in the high-risk group than in the low-risk group according to ssGSEA analysis. These results suggest a potential role for CTARSig in predicting TIME.

Advanced GC treatment is based on chemotherapy combined with neoadjuvant chemoradiotherapy, molecular targeted therapy, and immunotherapy. However, almost all patients with GC experience disease progression after treatment. After the second-line therapy, patients should be enrolled in clinical trials. Notably, the CTARSig-based risk stratification in this study contributed to the selection of individualised treatment regimens for patients with GC. Dasatinib is an orally administered short-acting inhibitor of multiple tyrosine kinases. One study showed that dasatinib and cisplatin enhance gastric cell death through the PI3K/AKT pathway, which is beneficial for patients with GC 42. Our study suggests that the high-risk group was more sensitive than the low-risk group.

Additionally, studies show that tipifarnib suppresses tumour aggressiveness in several malignancies and low-dose tipifarnib had antitumour effects only on HIF-1α-positive cells through the mTOR singling pathway, both *in vitro* and *in vivo* in GC model 43. In our study, the IC50 values indicated that low-risk patients were more sensitive to ipifarnib compared to high-risk patients. Furthermore, our data suggest that low-risk patients are more susceptible to chemotherapy and targeted drugs such as 5-fluorouracil, vinorelbine, doxorubicin, temozolomide, ruxolitinib, etoposide, gemcitabine, paclitaxel, and masitinib than high-risk patients.

In conclusion, this study aimed to identify and validate a novel cancer-testis antigen CTARSig for prognostication in STAD. Our results suggest that the CTARSig-based risk stratification can provide a basis for selecting individualised treatment options for patients with advanced GC. CTARSig, as developed in this study, can accurately predict the prognosis of patients with GC and has great potential as a novel biomarker. In addition, ELOVL4 may be a potential therapeutic target for GC, and the molecular mechanism regulating the malignant biological behaviour of tumour cells deserves further investigation.

## Supplementary Material

Supplementary figure.

## Figures and Tables

**Table 1:** Clinicopathological indicators of individuals in the TCGA-STAD cohort

Clinicopathological indicators	Type	TCGA-STAD cohort (n = 371)
**Age**	<=65	163 (43.94%)
	>65	205 (55.26%)
	Not available	3(0.80%)
**Gender**	Female	133 (35.85%)
	Male	238(64.15%)
**Grade**	G1	10 (2.70%)
	G2	134 (36.12%)
	G3	218 (58.76%)
	Not available	9 (2.42%)
**TNM-Stage**	Stage I	50 (13.48%)
	Stage II	111 (29.92%)
	Stage III	149 (40.16%)
	Stage IV	38 (10.24%)
	Not available	23 (6.20%)
**T-Stage**	T1	18 (4.85%)
	T2	78 (21.02%)
	T3	167 (45.01%)
	T4	100 (26.95%)
	Not available	8 (2.16%)
**N-Stage**	N0	108 (29.11%)
	N1	97 (26.15%)
	N2	74 (19.95%)
	N3	74 (19.95%)
	Not available	18 (4.85%)
**M-Stage**	M0	328 (88.41%)
	M1	25 (6.74%)
	Not available	18 (4.85%)

**Table 2:** Cancer-testis antigen-related signature in STAD

CTA-related genes	Coef	HR	*p*-value
MAGEA3	0.010700239	1.107	0.048
MAGEA11	0.167350315	1.415	0.009
THY1	0.097880794	1.238	0.010
SAGE1	0.100101094	1.229	0.048
PLAC1	0.158666022	1.446	0.029
TFDP3	0.475956093	2.785	0.014
ANGPT2	0.073519232	1.210	0.042
DNMT1	-0.063247504	0.693	0.022
ELOVL4	0.098339527	1.397	0.004
EZH2	-0.12369429	0.741	0.016
CHEK1	-0.018454819	0.797	0.048

HR, hazard ratio; Coef, Coefficient.
