# Supplementary material for: Identification and Validation of a Cancer-Testis Antigen-Related Signature to Predict the Prognosis in Stomach Adenocarcinoma
Source: J Cancer. 2024 May 11;15(11):3596–611. doi: 10.7150/jca.91842 (PMC11134429; doi:10.7150/jca.91842)
Supplement: Supplementary file 1 — Supplementary figure. [file jcav15p3596s1.pdf]

Supplementary Material

Supplementary Figures

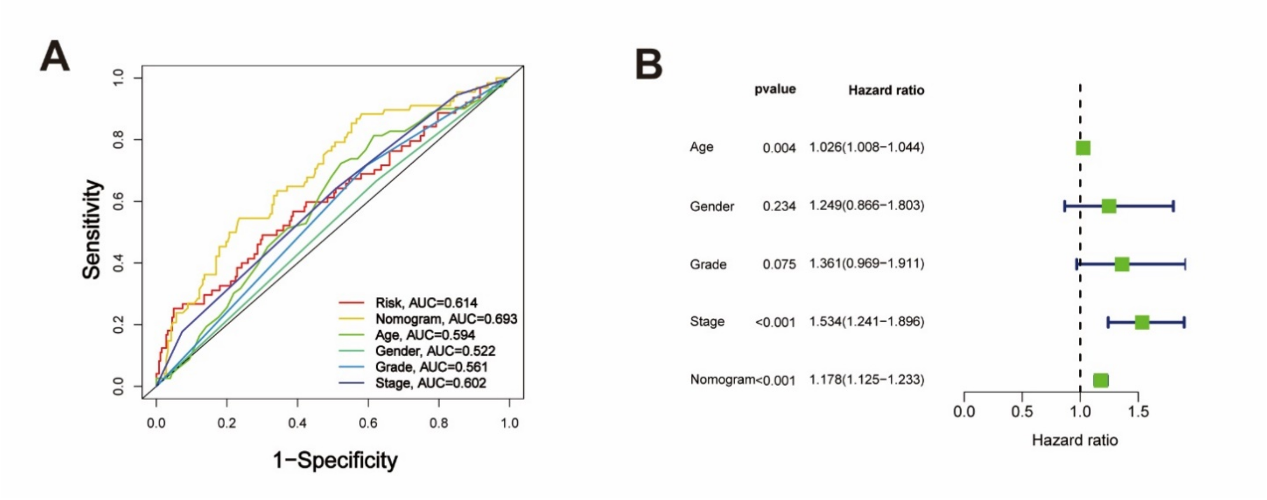

Figure S1 Assessment of the Nomogram in STAD. (A) ROC curves for the nomogram. (B) Forest plot for uni-Cox regression.
